# Challenges and solutions for upstream processing of complex biologics

**DOI:** 10.1093/abt/tbag008

**Published:** 2026-03-09

**Authors:** Shuya Xu, Yunlong Wang, Yawen Yu, Felix Zhu, Ying Li

**Affiliations:** Department of Antibody Process Development, GenScript ProBio Biotechnology Co., Ltd, Nanjing 21100, China; Department of Antibody Process Development, GenScript ProBio Biotechnology Co., Ltd, Nanjing 21100, China; Department of Antibody Process Development, GenScript ProBio Biotechnology Co., Ltd, Nanjing 21100, China; Department of Antibody Process Development, GenScript ProBio Biotechnology Co., Ltd, Nanjing 21100, China; Department of Antibody Process Development, GenScript ProBio Biotechnology Co., Ltd, Nanjing 21100, China

**Keywords:** upstream processing, cell culturebispecific antibody, fusion protein, titer enhancement, quality improvement

## Abstract

Unlike conventional monoclonal antibodies, complex biologics—such as bispecific antibodies and fusion proteins—often face challenges including lower expression levels, higher mispairing rates, and greater sensitivity to culture conditions, which can collectively limit both titer and product quality. Leveraging process development case studies, this review systematically explores upstream bioprocessing strategies aimed at mitigating these challenges, with a focus on titer enhancement, lactate metabolism regulation, acidic charge variants control, reduction of aggregates and fragments, and glycosylation optimization. Finally, a perspective toward future upstream development strategies is discussed.

## Teaser

**Statement of Significance** Complex molecules present hurdles in expression, assembly, and posttranslational modification, thereby limiting titer and quality. Drawing on platform research data, this work outlines upstream approaches designed to mitigate these challenges.

## Introduction

The landscape of complex biologics, such as bispecific antibodies (bsAbs) and fusion proteins, has evolved significantly over the past few decades, transitioning from experimental constructs to clinically validated therapeutics with expanding applications in oncology, immunotherapy, and beyond. The upstream process development for complex biologics is confronted with multifaceted technical and operational challenges stemming from molecular complexity, the dynamic nature of cell culture processes, and the stringent robustness requirements during scale-up. Current research and industrial practice highlight several interconnected difficulties.

For instance, in upstream process development, a primary challenge lies in achieving correct chain pairing and assembly. For bsAbs employing formats such as knob-into-hole, mispairing of heavy and light chains can lead to significant levels of product-related impurities—including homodimers and misassembled heterodimers—that are difficult to eliminate in downstream process [1]**.** Research on reducing mismatches mainly focuses on rational design at the molecular level. CrossMab technology effectively ensures correct pairing while maintaining antigen binding fidelity through domain exchange between heavy and light chains [2, 3]**.** The Duobody technique is a Fab arm exchange under reduced conditions and can also produce high-purity IgG-like bsAbs without gene fusion. In addition to molecular engineering, optimizing plasmid transfection ratios can reduce the proportion of homodimers and improve the purity of bsAbs [4]**.** Together, these methods enhance purity, yield, and manufacturability of therapeutic bsAbs.

Additionally, these molecules are often prone to aggregation and degradation, which can originate from exposed hydrophobic patches or charge imbalances introduced by fusion domains or engineered interfaces [5]. Aggregation not only compromises product quality but can also reduce viable titers and complicate purification. For instance, immunocytokines and Anticalin®-IgG fusions (e.g. Mabcalin™) demand meticulous cell line selection and process optimization to strike a balance between high yield and low aggregate formation, frequently integrating downstream purification data into upstream clone selection to identify optimal producing clones [5, 6].

Expression level also poses a major hurdle. Many bsAbs and fusion proteins are classified as “difficult-to-express” due to subunit imbalance, codon usage, or metabolic burden on the host cell [7]. In some extreme culture scenarios, such as too many reactive oxygen species (ROS), it can lead to inefficient glucose metabolism, with most of the glucose consumed converted to lactate and rarely into the tricarboxylic acid (TCA) cycle, which can also lead to reduced protein production. Holistic approaches—including signal peptide optimization, vector design, temperature shifts, and high-density seeding—have been employed to boost titers from <1 g/l to over 10 g/l in some cases [7]. Host cell engineering, such as constitutive expression of productivity-enhancing miRNAs (e.g. miR-557), has also shown promise in improving both clone selection success rates and final titers, particularly for unstable molecules [8].

Glycosylation profiles and charge heterogeneity are highly sensitive to process parameters such as media composition, pH, temperature, and dissolved oxygen [9, 10]. Glycosylation affects key functional attributes including protein folding, protease resistance, thermal stability, serum half-life, immunogenicity, receptor binding affinity, and effector functions such as antibody-dependent cellular cytotoxicity (ADCC) [11, 12]. Acidic and basic charge variants of antibodies arise primarily from posttranslational modifications (PTMs), such as deamidation, oxidation, sialylation, C-terminal lysine retention, and N-terminal pyroglutamate formation. These variants can influence antibody structure, stability, and biological function, although the extent of impact varies significantly across molecules and modification types.

In summary, upstream development for bsAbs and fusion proteins demands integrated molecular design, robust cell line development, and adaptive bioprocess strategies to address chain mispairing, low expression, aggregation, and atypical PTMs—often requiring feedback loops between upstream and downstream process steps to ensure manufacturability [3, 13, 14]. This article provides a comprehensive review of upstream strategies aimed at enhancing production efficiency and product quality. The discussion is structured around several critical aspects, including the improvement of titer, regulation of lactate metabolism, control of charge heterogeneity, minimization of fragment and aggregate formation, and optimization of glycosylation. Each strategy is illustrated with practical examples from our industrial practices, highlighting how these approaches can be effectively integrated into upstream processes to address common challenges and achieve more robust and scalable bioproduction.

## Solutions for challenging cell culture process

The Chinese hamster ovary (CHO) cell culture process comprises a series of complex biochemical events—such as protein synthesis, secretion, and PTMs—all of which are highly sensitive to the culture environment. Factors including pH, temperature, ROS, nutrient availability, and the accumulation of metabolic byproducts can significantly impact both the titer and quality of the target protein.

This section consolidates effective control strategies to address typical challenges in upstream cell culture process.

## Improving titer

Titer is defined as the product of the integral of viable cell density (IVCD) and the specific productivity (qP), representing the overall volumetric productivity of a cell culture process. Accordingly, strategies to increase titer focus on enhancing either cell growth (IVCD), per-cell productivity (qP), or both [15, 16].

**Figure 1:**
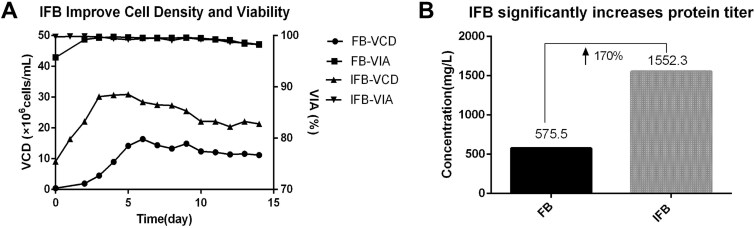
Comparison of FB and IFB cultures. (A) IFB significantly enhances viable cell density compared to FB culture. (B) The titer improvement by IFB is achieved through enhanced cell density.

Process intensification technologies are pushing CHO cell cultures toward their maximum viable density. The intensified fed-batch (IFB) strategy is central to this advancement, differing from conventional fed-batch (FB) in both setup and output. While conventional FB starts with a low seed density (~0.5 × 10^6^ cells/ml) and peaks around 20 × 10^6^ cells/ml, IFB employs a much higher seed density (~5–20 × 10^6^ cells/ml). Supported by precision feeding strategies, IFB not only shortens the production duration but also sustains significantly higher peak cell densities, thereby pushing the limits of volumetric productivity. We established an IFB process for a fusion protein, in which an N-1 perfusion bioreactor was used to elevate the seeding density from 0.5 × 10^6^ to 10 × 10^6^ cells/ml, and feeding was initiated on the day of inoculation. These strategies resulted in a 138% increase in the final IVCD (Fig. 1A), thereby tripling the product titer from 575.5 to 1552.3 mg/l (Fig. 1B) without compromising critical quality attributes (CQAs) such as protein purity.

**Figure 2:**
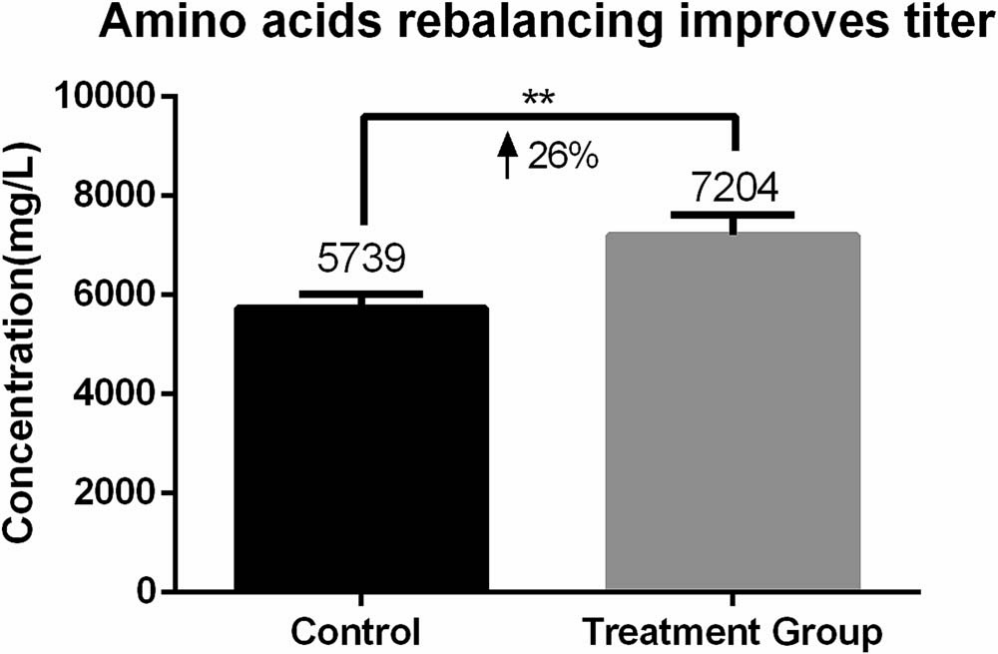
Improving titer by rebalancing AAs concentrations.

Chemically defined media comprise over 50 components, making it challenging to assess the impact of each on process performance and product quality. Amino acids stand out as fundamental nutrients, as they not only promote cell growth and productivity as nitrogen sources and protein building blocks but also regulate key metabolic pathways. In recent years, several studies have shown that optimizing amino acids significantly improves cell viability, extends culture cycles, and increases target protein titers [17]. For example, in a study of bevacizumab-producing cell lines, the Plackett–Burman design combined with the response surface method was used to optimize the amino acid combination, which ultimately increased the antibody titer by 70%. The supplementation of specific amino acids was confirmed to directly promote metabolic efficiency. For example, valine addition not only increases erythropoietin (EPO) production by 25%, but also simultaneously reduces ammonia and lactate accumulation by 23%–26% [18]. Similarly, synergistic supplementation of serine, cysteine, and tyrosine has been shown to improve metabolic profiles and enhance antibody titer, especially when available as tripeptides, and to overcome solubility limitations [19]. Notably, depletion of nonessential amino acids such as asparagine and glutamine triggers cell cycle arrest, while cysteine deficiency directly impairs proliferation and productivity [20]. Some researchers have used metabolomics to investigate the correlation between 97 metabolites and both protein qP and cell growth. It was found that metabolites significantly correlated with qP and growth are enriched in the TCA cycle, Ala, Asp, and Glu metabolism pathways [21]. Since amino acids can enter the TCA cycle through different intermediates, amino acids were significantly correlated with qP and growth. As shown in Fig. 2, our own practice showed that adjusting amino acid contents can significantly improve the titer by increasing qP (from 19 to 32 pg/cell/day).

In mammalian cell culture, controlling pCO_2_ and osmolality is an important strategy for optimizing cell density and product yield. Research shows that high pCO_2_ (such as above 150 mmHg) usually inhibits the cell growth rate. For example, when the pCO_2_ of CHO cells rises from 50 to 150 mmHg, the specific growth rate decreases by approximately 9% [22]. At 250 mmHg, growth inhibition can reach 30% [23]. It is recommended to control the pCO_2_ below 120 mmHg in large-scale production. Effective CO_2_ stripping can be achieved by increasing the agitation and sparger rate or through utilizing a frit sparger [24]. An increase in osmolality also inhibits cell proliferation, such as a 60% reduction in growth rate at 450 mOsm/kg [22]. However, the effects of both on product synthesis are complex: although high osmolality inhibits growth, it is often accompanied by an increase in qP, e.g. at 460–500 mOsm/kg, antibody titers do not decrease, as the increase in qP compensates for the loss of cell density. Another study has shown that antibody production can be increased by nearly 90% in a 350 mOsm/kg environment [25]. Several other common strategies for increasing cell density and qP are listed in Table 1.

**Table 1:** Strategies to enhance IVCD and qP

Category	Specific methods to enhance IVCD	Specific methods to enhance qP
Process parameters	Temp. and pH shift Control of pCO_2_ and osmolality Implementation of IFB or perfusion	Temp. shift Control of pCO_2_ and osmolality
Media components	Optimization of key media components, including: Amino acids Trace elements and inorganic acids (e.g. selenite, Fe, Zn, Na) Nucleosides (e.g. cytidine, uridine, thymidine)	Optimization of key media components, including: Amino acids TCA cycle intermediates Antioxidants Trace elements

## Lactate regulation

Lactate is a major byproduct of CHO cell metabolism, primarily generated from the glycolytic conversion of glucose to pyruvate followed by its reduction via lactate dehydrogenase (LDH) [26]. In FB culture, lactate accumulation typically follows distinct phase-dependent patterns: an initial increase during exponential growth, often followed by partial or complete consumption in the mid-to-late culture phase [27]. A major challenge arises when lactate is not efficiently consumed or when it undergoes a secondary rise at later stages due to nutrient depletion, accumulation of inhibitory byproducts, or reduced mitochondrial efficiency [28, 29]. Persistent lactate buildup leads to medium acidification, impaired cell growth, and compromised product yield and quality [30].

To address the challenge of lactate accumulation, it is important to consider the underlying metabolic mechanisms. In CHO cells, pyruvate serves as a central metabolic node: it can be reduced to lactate in the cytosol via LDH, transaminated to alanine, or transported into mitochondria for complete oxidation through the TCA cycle and electron transport chain [27]. Excessive glycolytic flux leads to pyruvate accumulation, which is predominantly converted to lactate, resulting in high extracellular lactate levels. The key to mitigating this issue lies in directing pyruvate away from lactate formation and toward oxidative metabolism. Accordingly, lactate-control strategies can be conceptualized within two complementary frameworks: (i) suppression of excessive glycolytic flux to limit pyruvate formation, and (ii) enhancement of oxidative metabolism to channel pyruvate through the TCA cycle and electron transport chain [26, 27].

Representative strategies aligned with these mechanisms are summarized in Table 2. For example, glucose limitation directly suppresses glycolytic flux by restricting substrate availability, thereby lowering pyruvate and subsequent lactate production [26, 27]. Temperature shift slows overall metabolic rates, favoring mitochondrial oxidation of pyruvate [26, 27]. Supplementation with TCA intermediates (such as pyruvate, α-ketoglutarate, or malate) provides substrates to replenish the TCA cycle and boost mitochondrial flux, which enhances pyruvate entry into the TCA cycle and alleviates pyruvate accumulation, consequently suppressing lactate production [29]. Copper supplementation serves as a cofactor for cytochrome c oxidase, enhancing respiratory efficiency and reducing lactate accumulation [31]. Optimized feeding strategies—particularly dynamic or on-demand feeding—maintain stable glucose and nutrient levels, preventing transient metabolic stress that could otherwise trigger glycolytic overflow and secondary lactate spikes [32]. Collectively, these interventions modulate the balance between cytosolic and mitochondrial metabolism, supporting a more oxidative and energy-efficient phenotype in CHO cell culture [26, 27].

**Table 2:** Lactate metabolism control strategy in CHO cell culture

Mechanism	Solution strategies
Reduce glycolysis	Glucose limitation Temperature shift (e.g. 36.5 → 31°C) pH modulation Optimized feeding strategy
Enhance oxidative metabolism	Supplementation with TCA intermediates (e.g. pyruvate, α-KG, malate, succinate) Copper supplementation in basal media Temperature shift (e.g. 36.5 → 31°C) Optimized feeding strategy Controlled pCO_2_ levels (30–120 mmHg)

Note: Several strategies, such as temperature shift and optimized feeding, contribute to both mechanisms by simultaneously limiting glycolysis and enhancing oxidative metabolism, thereby achieving a more balanced metabolic state.

**Figure 3:**
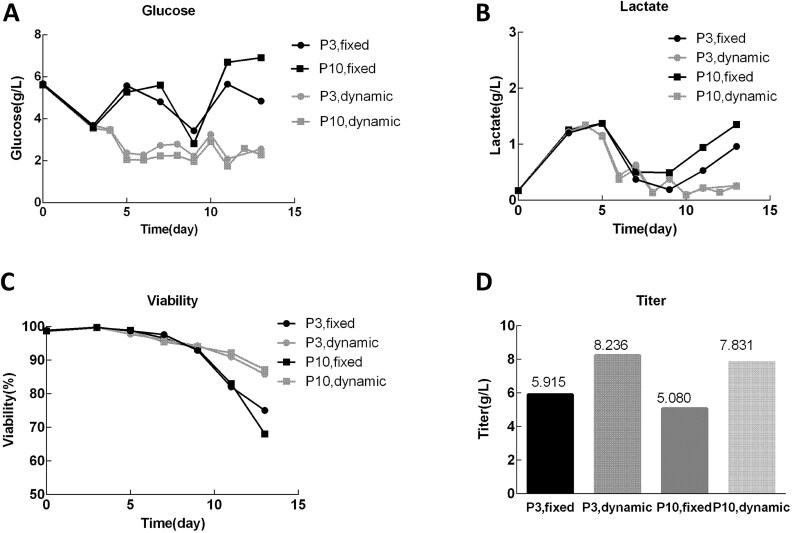
Comparative analysis of cell growth, metabolism, and expression under fixed and dynamic feeding strategies. (A) Dynamic feeding maintained stable glucose levels throughout the culture. (B) and (C) Dynamic feeding prevented late lactate rebound and improved cell viability. (D) Dynamic feeding achieved higher titer across two passages.

An IgG1 production study based on our internal experimental data highlights the practical benefits of feeding strategy optimization. In this case, glucose was used as an indicator to drive a dynamic feeding strategy, in which glucose was supplied exclusively via the feed, and feed input was adjusted daily according to glucose consumption to restore glucose to defined target levels (approximately 5 g/l during Day 2–Day 6 and 7 g/l during Day 7–Day 12). In contrast, under a fixed-ratio feeding regime, nutrients were supplied at predefined ratios, and glucose was additionally adjusted to 8–10 g/l by separate glucose supplementation on alternating days. As shown in Fig. 3A, glucose concentrations represent residual glucose levels measured prior to daily feeding. Compared with fixed-ratio feeding, the dynamic feeding strategy effectively suppressed lactate accumulation and mitigated late-stage buildup (Fig. 3B). This benefit cannot be attributed solely to lower glucose levels; rather, it arises from the demand-driven nature of dynamic feeding, whereby increased glucose consumption automatically leads to increased feed addition and proportional cosupply of other nutrients. Consistent with this mechanism, dynamic feeding resulted in a higher overall feed ratio (42.8%) compared with the fixed-ratio feeding strategy (35%). As a result, culture robustness was improved, sustaining higher cell viability (Fig. 3C), and productivity was enhanced, with titers increasing from 5.9 to 8.2 g/l in passage 3 and from 5.1 to 7.8 g/l in passage 10 (Fig. 3D). In addition, process consistency across different cell passages was significantly improved.

## Reducing acidic variants

Antibodies present various forms of heterogeneity in size, charge, and other aspects during production due to enzymatic reactions or spontaneous degradation and modification. It can be chemically modified through several different mechanisms, including oxidation, deamidation, isomerization, and fragmentation, resulting in various charge isomers and changes in their isoelectric point (pI) values.

Chemical and enzymatic modifications, such as deamidation and sialylation, lead to an increase in the net negative charge on the antibody and a decrease in the pI value, respectively, resulting in the formation of acidic variants. Another mechanism for the generation of acidic variants is the formation of various types of covalent adducts, such as glycation, which is the reaction of glucose or lactose with the primary amines of antibody lysine residues in glucose-rich media or during storage.

Charge heterogeneity may have potential effects on antibody efficacy, immunogenicity, and clearance. There is evidence that a change in the pI of an antibody by only one or more units can produce significant differences in the pharmacokinetics (PK) of the intact antibody [33]. Research has shown that acidic variants may weaken their binding to FcRn in an acidic endosomal environment due to increased surface negative charge, resulting in faster lysosomal degradation and clearance [34]. Deamidation in the complementarity-determining regions will significantly reduce the binding of the antibody to the antigen, thereby decreasing the antibody efficacy. Aggregates are sometimes detected within the acidic species, and aggregation has been shown to increase immunogenicity [35]. Sialic acid modification generally does not affect the binding to the antigen, but a few studies have shown that sialic acid modification can lead to a decrease in ADCC activity and a prolongation of half-life [34].

As shown in Table 3, reducing the acidic species is mainly achieved by lowering or scavenging ROS, decreasing the proportion of deamidation, reducing the reduction of antibody disulfide bonds, and the formation of cysteine [35, 36]. Lowering pH can slow down the deamidation of Asn and reduce the formation of Asp and isoAsp, both of which reduce the pI of antibodies [37]. Oxidation of Trp residues contributes to the generation of acidic variants. This reaction can be suppressed by adding free Trp to the medium, which functions as a competitor of the Trp site of the antibody, thereby lowering the acidic species. Mn is a key cofactor of manganese superoxide dismutase, which can catalyze the conversion of superoxide anion to hydrogen peroxide, thereby alleviating intracellular oxidative stress and potentially reducing the formation of acidic species [38]. Increasing basic amino acids inhibits the removal of terminal lysine, systematically increases the net positive charge of the antibody, and makes more molecules migrate to the main or basic species, thereby relatively diluting the proportion of acidic species [39].

**Table 3:** Solutions for reducing protein acidic variants

Mechanism	Specific approach
Reducing protein deamidation	Lower pH
Alleviating protein oxidation	Supplementing Trp in basal media
	Supplementing Mn^2+^ in basal media
	Reduce the addition amount of FeedB (high-concentration feeding)
	Substituting Fe^2+^ with ferric ammonium citrate
	Supplementing bioflavonoids in late stage of cell culture
Minimizing unpaired cysteines and cysteinization	Reducing the addition amount of FeedB (high-concentration feeding)
	Supplementing Cu^2+^ and cystine in basal media
Increasing positive charge	Supplementing Arg/Lys/His/Orn in basal media

**Figure 4:**
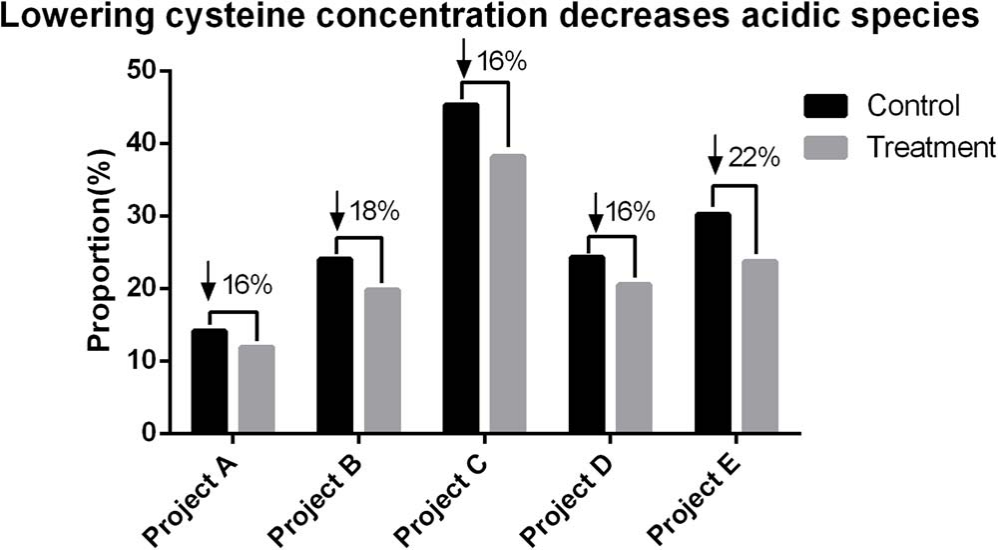
Reducing acidic species by lowering cysteine concentration in different projects.

Excessively high concentrations of cysteine can lead to the reduction of disulfide bonds and cysteinization of antibodies. Cysteine can also lead to the production of ROS, thereby causing the oxidation of antibodies. All these modifications will introduce negative charges, causing an increase in acidic peak in ion-exchange chromatography [40]. As shown in Fig. 4, our results indicated that lowering cysteine concentration can reduce the acidic species to varying degrees.

## Reducing aggregation and fragmentation

Protein aggregates are high molecular weight (HMW) species consisting of multiple protein molecules associated through covalent or noncovalent interactions. Aggregate species is considered a CQA as it can impact product potency, stability, and PK, and may elicit undesired immunogenic responses *in vivo* [41].

Aggregate formation is typically classified into covalent aggregation and noncovalent aggregation. Covalent aggregation involves the formation of irreversible covalent cross-links between protein molecules. Free cysteine thiols in antibodies can catalyze the scrambling or incorrect formation of intermolecular disulfide bonds (S–S), linking monomers together [42]. Noncovalent aggregation is primarily driven by the exposure of hydrophobic regions on the protein's surface. Stressors such as heat, agitation, shear stress, or exposure to interfaces (e.g. air–liquid, container surface) can partially unfold the protein. The exposed hydrophobic patches then interact with similar regions on other molecules, leading to their association [43].

Fragments are low molecular weight species resulting from the cleavage of an intact antibody into smaller pieces, such as Fab, Fc, or single chains. During the cell culture process, enzymes such as proteases found in host cell proteins (HCPs) can cleave specific peptide bonds in antibodies, particularly in the flexible hinge region, generating Fab and Fc fragments. Fragment formation also involves disulfide bond reduction and scrambling. Free thiols (–SH) or reducing agents (originating from cell culture media or impurities) can reduce the interchain disulfide bonds that hold the heavy and light chains together. This leads to the generation of half-antibodies or free light/heavy chains [44].

Table 4 presents the mechanisms for aggregate and fragment formation, as well as corresponding mitigation strategies.

**Table 4:** Strategies to reduce aggregate and fragment

Aggregate/fragment	Mechanism	Strategies
Aggregate	Hydrophobic interaction	Lowering titer
		Supplementing glycerol and DMSO in basal media
		Reducing temp.
		Supplementing NaCl in basal media
Fragment	Specific peptide bond cleavage	Lower temp.
		Shorter culture duration
		Adopting perfusion process
		Supplementing metal ion chelators in basal media
Fragment and aggregate	Disulfide bond reduction and scrambling	Increasing DO
		Supplementing redox substances (e.g. cysteine/cystine/copper) in basal media
		Supplementing metal ion chelators in basal media

A reduction in temperature, for instance, has been identified as a prevalent and efficacious approach to mitigate aggregation and fragmentation. It has been demonstrated by a number of studies that a reduction in temperature can result in an enhancement of protein PTM capacity and a reduction in aggregation levels [45]. Knight et al. reported that a reduction in temperature can retard the process of mRNA translation, enhance cell viability, curtail the secretion of various protein hydrolases into the extracellular environment, and further decrease fragmentation level [46].

**Figure 5:**
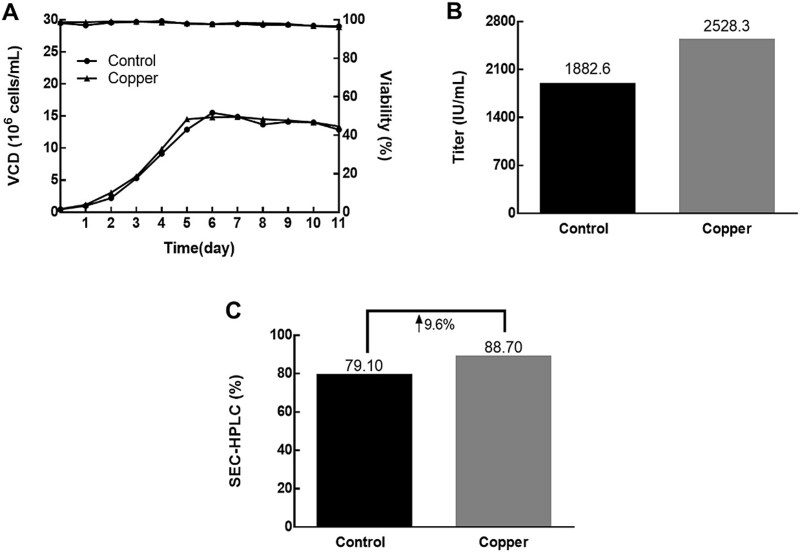
Investigating the impact of copper supplementation on protein SEC and cell growth. (A) The addition of copper did not affect the cell growth status. (B) and (C) Addition of copper increases the titer and decreases the proportion of aggregates, thereby enhancing the SEC main peak.

In an in-house coagulation factor VIII production study, a low-purity challenge arose as a product with insufficient monomer may be less effective and potentially cause an immune response [47]. To address this issue, copper ions were employed as a redox substance to reduce the formation of aggregates/fragments resulting from disulfide bond reduction and scrambling. It has also been reported that adding copper ions to late-stage cultures can reduce free thiol content and HMW species [48, 49]. In this case, adding 10 μM copper ions to the basal medium resulted in a significant 10% increase in the main size exclusion chromatography (SEC) peak, rising from 79.10% to 88.70% (Fig. 5A), with no adverse effect on cell growth or titer (Fig. 5B and C).

**Figure 6:**
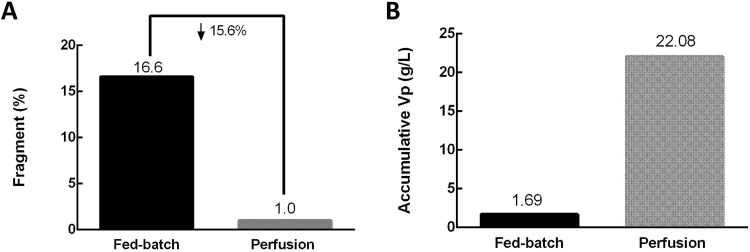
(A) Compared with FB, perfusion significantly reduces the proportion. (A) The perfusion process significantly increased the titer.

A case study involving a fusion protein consisting of an mAb and a SpyTag protein revealed a fragmentation issue. This results in the loss of key amino acids in the fused protein sequence and an undesired loss of binding potency. In this case, the perfusion process was adopted to address this issue. This process continuously removes cell waste and spent medium while replacing nutrients and carbon sources with fresh medium, thereby maintaining a constant environment throughout the entire production phase. The kinetics of all processes in the reactor remain unchanged over time, including those related to impurities and PTMs [50]. This approach can reduce the accumulation of waste metabolites and cellular content released due to cell death and mitigate intracellular ROS accumulation, which alleviates glutathione oxidation that occurs in FB processes. As a result, a favorable intracellular redox environment is maintained, which can attenuate mitochondrial dysfunction and endoplasmic reticulum (ER) stress [50, 51]. In our study, the perfusion process significantly decreased the fragmentation proportion by 15.6%, from 16.6% to 1.0%, (Fig. 6A) and demonstrated high productivity (22.08 g/l), significantly exceeding that of the FB process (1.69 g/l) (Fig. 6B).

## Glycosylation modulation

Glycosylation is widely recognized as one of the most critical CQAs of therapeutic proteins, particularly antibodies and Fc-fusion proteins [52]. It influences biological activity, serum half-life, structural stability, and immunogenicity. Even subtle variations in glycan structures—such as the extent of fucosylation, galactosylation, sialylation, and high-mannose content—can significantly alter clinical efficacy and safety [52]. Consequently, achieving consistent and controllable glycosylation has become a central objective in upstream bioprocess development.

N-linked glycosylation in CHO cells follows a well-orchestrated biosynthetic pathway initiated in the ER and completed in the Golgi apparatus. In the ER, a preassembled oligosaccharide is transferred to nascent polypeptides, followed by trimming steps that ensure proper protein folding and quality control. The partially processed glycan then undergoes sequential modification in the Golgi through the concerted action of glycosidases and glycosyltransferases, resulting in a diverse repertoire of complex, hybrid, and high-mannose structures. The final glycan profile is dictated not only by the expression and activity of glycosylation enzymes but also by the intracellular availability of nucleotide sugar donors (e.g. UDP-galactose, CMP-sialic acid, GDP-fucose) and essential metal cofactors [53].

Although the glycosylation machinery is intrinsic to the host cell, it remains highly sensitive to the extracellular environment. Process parameters—including temperature, pH, dissolved oxygen, pCO_2_, and osmolality—as well as the composition of basal media and feeds, have all been reported to influence glycosylation outcomes by modulating enzyme activities and precursor supply [54]. These extrinsic factors therefore provide practical levers for modulating glycan structures, and a range of representative upstream strategies has been developed to steer glycosylation toward desired quality profiles (Table 5).

**Table 5:** Glycosylation modulation strategy in CHO cell culture

Glycosylation type	Goal	Process parameter control	Media/additive optimization
Mannosylation	Increase	High osmo. Low temp. Prolong culture duration	Kifunensine High NH_4_^+^ Tagatose, sucrose, mannose
	Decrease	Low osmo. High temp. Shorten culture duration	Manganese ManNAc
Galacosylation	Increase	High DO Low osmo. High pH	Uridine Manganese Galactose
	Decrease	High osmo. High pCO_2_ Low pH	Gln/Asn/High NH_4_^+^
Afucosylation	Increase	Low pH	2-F-peracetyl fucose Mycophenolic acid Kifunensine
	Decrease	High pH	Fucose
Sialylation	Increase	High DO Low osmo. High pH High temp. Shorten culture duration	Uridine Manganese Galactose *N*-Acetylmannosamine Copper Dexamethasone
	Decrease	Low temp. High osmo. Extend culture duration	Sodium butyrate Gln/Asn/High NH_4_^+^

**Figure 7:**
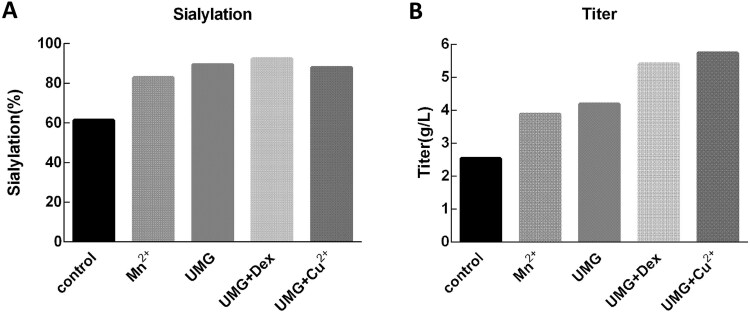
Effects of additive combinations on sialylation and titer. (A) All four additive combinations resulted in a significant increase in the sialic acid ratio. (B) Beyond elevating sialic acid levels, they also significantly enhanced protein titer.

In our own Fc-fusion protein case, the major challenge was insufficient terminal sialylation, limiting pharmacokinetic potential. To address this, cultures were supplemented with different additives that target key points in the sialylation pathway (Fig. 7A), which can also increase protein yield to varying degrees (Fig. 7B). Specifically, Mn^2+^, an essential cofactor for galactosyltransferase and sialyltransferase [55], increased sialylation to 82.9% and titer to 3.88 g/l by enhancing the enzymatic activity required for terminal galactosylation and subsequent sialylation. Uridine, manganese, and galactose (UMG) further improved sialylation (89.3%) and titer (4.19 g/l) by simultaneously increasing the intracellular pool of nucleotide sugar donors (UDP-galactose and CMP-sialic acid) and providing Mn^2+^ to support glycosyltransferase function [56]. The combination of UMG with dexamethasone achieved the highest sialylation (92.3%) while maintaining strong productivity (5.41 g/l), as dexamethasone can further stimulate glycosyltransferase expression [57]. UMG with copper ion also supported high sialylation (88.0%) and gave the highest titer (5.74 g/l), likely because Cu^2+^ acted as a competitive inhibitor of sialidase, preventing the removal of terminal sialic acids while UMG supplied nucleotide sugar donors and Mn^2+^ supported transferase activity [58]. As indicated previously, Mn^2+^ can enhance the titer. This is due to its function as a cofactor for mitochondrial superoxide dismutase 2, which scavenges mitochondrial ROS [59]. In addition, dexamethasone can also increase titer; by activating glucocorticoid receptors, dexamethasone upregulates the expression of antiapoptotic genes and glutathione reductase, thereby promoting cell viability and improving yield [60].

**Figure 8:**
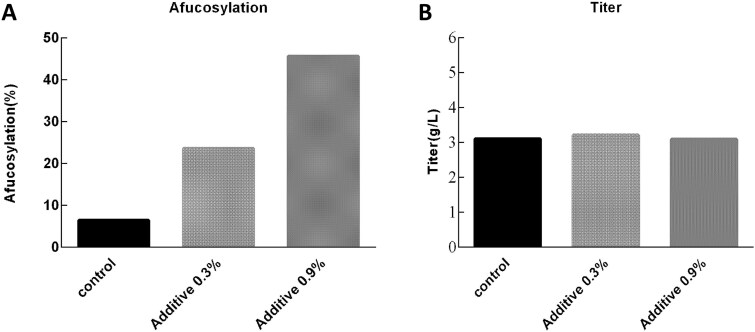
Effects of additive on afucosylation and titer. (A) Addition of 0.3% or 0.9% additive significantly increased afucosylation from 6.45% in the control to 23.62% and 45.59%, respectively. (B) Titer remained largely unaffected across all conditions.

In our IgG1 project, the major goal was to increase afucosylation, which is critical for enhancing ADCC. To address this, cultures were supplemented with a specific additive containing fucose analogs that target key steps in the fucosylation pathway. This additive likely increases afucosylation by competitively inhibiting fucosyltransferase activity, thereby reducing core fucosylation and enhancing the proportion of afucosylated Fc glycans. As shown in Fig. 8, adding 0.3% of the additive increased afucosylation from 6.45% in the control to 23.62% without negatively affecting titer, while a higher concentration of 0.9% further elevated afucosylation to 45.59%.

## Summary and outlook

The development of upstream processes for complex biologic therapeutics, including monoclonal antibodies, bsAbs, and fusion proteins, represents a critical and challenging phase in the biopharmaceutical industry. The pursuit of a robust, scalable, and economically viable manufacturing process is often hampered by a myriad of interconnected challenges that can impact both product yield and quality.

This review provides a landscape analysis of the current challenges and solutions in upstream process development for complex biologics. It begins by synthesizing persistent issues such as suboptimal product titer, high levels of aggregates, fragments, and acidic charge variants, and difficulties in controlling glycosylation. A dedicated analysis of lactate metabolism is also provided, highlighting the emerging trend of lactate rebound during the late phases of cell culture—a phenomenon that critically undermines process robustness and scalability.

In response to these challenges, the second part of the review consolidates a comprehensive set of advanced strategies, spanning from media formulation and feeding strategies to process parameter control. The practical value of this work is underscored by the fact that these strategies have been successfully implemented and scaled within our platform development projects, demonstrating strong applicability and facilitating the development of more robust and scalable upstream processes.

The integration of artificial intelligence (AI) and advanced process analytical technology (PAT) is poised to fundamentally transform upstream bioprocessing from an empirically driven practice into an intelligently orchestrated, data-centric endeavor.

In the realm of AI, digital twin and hybrid modeling are being increasingly harnessed for the precise regulation of mammalian cell culture processes. By integrating cell culture monitoring data with mechanism-based modeling and data-driven modeling, digital twins enable the prediction of various key parameters, such as viable cell density, metabolic profiles, product titer, and more [61, 62]. In silico modeling simulates complex interactions among cell behavior, nutrient transport, mechanical forces, and metabolic dynamics to provide predictive insights into cell culture processes, reducing experimental costs and accelerating process development [63, 64].

Furthermore, real-time monitoring of CQAs is increasingly feasible through advanced PAT tools such as *in situ* Raman spectroscopy and soft sensors. Raman spectroscopy combined with the construction of automated machine learning models has enabled simultaneous monitoring of over 100 metabolites, promoting rapid process optimization [65]. In addition, the PAT-on-a-chip microfluidic platform now enables online multiattribute analysis, including glycan analysis and HCP quantification, with performance comparable to traditional offline methods, thereby accelerating data-driven decision-making [66]. Real-time glucose control via Raman feedback has also been shown to reduce product glycation levels from ~9% to 4%, directly improving CQAs [67]. When embedded within closed-loop control architectures, PAT-generated data can drive automated process adjustments, supporting intensified feeding regimes, mitigating process upsets and laying the groundwork for real-time release in upstream process.

Future development will focus on building self-optimizing bioreactor systems capable of dynamically adapting to process variability and maintaining optimal performance across scales. Key to this vision will be the creation of standardized, interoperable data ecosystems, improved model interpretability, and the validation of AI-PAT frameworks under regulatory scrutiny—ultimately enabling a more predictive, robust, and continuous upstream manufacturing paradigm.

## Data Availability

The authors confirm that the data supporting the findings of this study are available within the article.
